# Spatio-Temporal Patterns in Rhizosphere Oxygen Profiles in the Emergent Plant Species *Acorus calamus*

**DOI:** 10.1371/journal.pone.0098457

**Published:** 2014-05-27

**Authors:** Wang Wenlin, Han Ruiming, Wan Yinjing, Liu Bo, Tang Xiaoyan, Liang Bin, Wang Guoxiang

**Affiliations:** 1 Jiangsu Key Laboratory of Environmental Change and Ecological Construction, College of Geographical Science, Nanjing Normal University, Nanjing, China; 2 Nanjing Institute of Environmental Sciences, Ministry of Environmental Protection, Nanjing, China; 3 Jiangsu Environmental Engineering Consulting Center, Nanjing, China; 4 School of Geography Science, Nantong University, Nantong, China; Instituto de Biología Molecular y Celular de Plantas, Spain

## Abstract

Rhizosphere oxygen profiles are the key to understanding the role of wetland plants in ecological remediation. Though *in situ* determination of the rhizosphere oxygen profiles has been performed occasionally at certain growing stages within days, comprehensive study on individual roots during weeks is still missing. Seedlings of *Acorus calamus*, a wetland monocot, were cultivated in silty sediment and the rhizosphere oxygen profiles were characterized at regular intervals, using micro-optodes to examine the same root at four positions along the root axis. The rhizosphere oxygen saturation culminated at 42.9% around the middle part of the root and was at its lowest level, 3.3%, at the basal part of the root near the aboveground portion. As the plant grew, the oxygen saturation at the four positions remained nearly constant until shoot height reached 15 cm. When shoot height reached 60 cm, oxygen saturation was greatest at the point halfway along the root, followed by the point three-quarters of the way down the root, the tip of the root, and the point one-quarter of the way down. Both the internal and rhizosphere oxygen saturation steadily increased, as did the thickness of stably oxidized microzones, which ranged from 20 µm in younger seedlings to a maximum of 320 µm in older seedlings. The spatial patterns of rhizosphere oxygen profiles in sediment contrast with those from previous studies on radial oxygen loss in *A. calamus* that used conventional approaches. Rhizosphere oxygen saturation peaked around the middle part of roots and the thickness of stably oxidized zones increased as the roots grew.

## Introduction

Oxygen released by the roots of wetland plants can actively alter the micro-environmental parameters in the rhizosphere and subsequently affect various biogeochemical processes [1]. The roots of well-adapted wetland plants maintain a continuous downward flow of oxygen due to the formation of aerenchyma (spongy tissue between the cells that functions in gas exchange) and internal ventilation processes [2], [3]. Oxygen is proportionally consumed during root growth, but substantial amounts of oxygen are released into the rhizosphere, a phenomenon called radial oxygen loss (ROL). The released oxygen oxidizes the surrounding reduced substances and creates heterogeneous micro-niches around the root surface [4].

Oxygenation of the hypoxic rhizosphere raises the redox potential, which is beneficial to aerobic biogeochemical processes, such as acidogenesis, acetogenesis, methanogenesis, and denitrification [1], [5]. It is unfavorable to anaerobic processes, such as the production of organic acids and methane [5]. The oxic interface around the root surface also facilitates the formation of iron and manganese plaques on the root surface, which helps to immobilize heavy metals and phosphorus [6], [7], [8]. Consequently, intense processes of exchange, precipitation, and decomposition occur in the rhizosphere, enhancing the absorption and degradation of various contaminants [2], [9], [10].

Quantitative and dynamic information concerning the oxygen profiles in the rhizosphere can disclose the ecological role played by plant roots as they release oxygen. Due to the complex composition, structure, and properties of natural sediments, numerous investigations focusing on ROL in wetland plants have used artificial substrates, mainly agar medium, rather than *in situ* measurements of sedimentary oxygen profiles [11], [12], [13]. Three conventional techniques are cylindrical platinum electrode [14], titanium (III)-citrate [15], and anthraquinone radical anion methods [16]. Although the experimental setup is quite different from real sedimentary conditions, ROL assay in agar has proven that the net efflux of oxygen from different parts of roots varies and that the patterns are species specific [12]. The actual rhizosphere oxygen profile is comprehensively determined by the amount of oxygen released and by rhizosphere depletion [15].

Novel techniques based on microsensors have shed light on the direct monitoring of rhizosphere oxygen profiles in sediment [4], [5], [15], [17], [18], [19], [20]. These direct sedimentary assays have confirmed the existence of a stably oxidized rhizosphere that depends on the amount of oxygen released into the hypoxic soil, and that has varying thickness at different positions along the root [15], [20].

Detailed mapping of the rhizosphere oxygen dynamics has been a major challenge in rhizosphere research. In sediment, monitoring of rhizosphere oxygen is complicated by the variation and/or fluctuation in oxygen release over time along the entire root system [18], [21]. A novel technique using planar optodes allows fine mapping of rhizosphere oxygen conditions, providing both quantitative and dynamic information [4], [5], [18], [22]. However, this approach cannot follow a single root as it grows; and are only feasible to those roots which present in the testing zone. The diameter of the rhizobox [5], [19] is also too large to avoid capturing interactions among several closely positioned roots that may greatly differ in age and oxygen release capacity. The monitoring of semi-controlled root growth and spatio-temporal oxygen profiles is a good alternative which supply information about a selected single root during its entire period of growth.

The genesis of exodermis and formation of iron plaques as well as the aging of the root itself may induce pronounced changes in spatial patterns and the efflux rate of oxygen release from the roots [2], [23]. Though *in situ* determination of the rhizosphere oxygen profiles has been performed occasionally at certain growing stages within periods of days, comprehensive study on individual roots during weeks is still missing.

In the present study, we used a micro-optode sensor to investigate the spatio-temporal patterns of rhizosphere oxygen profiles in individually selected roots of the emergent plant species *Acorus calamus*. *A. calamus* is a perennial, obligate wetland herbaceous plant that is widely distributed in temperate and subtropical regions and that grows in seasonal and nonperiodic shallow waters [24]. It has been used in China for wastewater management in constructed wetlands, and has shown great potential for nutrient removal and heavy metal immobilization [25], [26]. Emergent plants have well-developed aerenchyma and root systems compared with submersed macrophytes, and are thought to release larger amounts of oxygen to sediment [17]. Using the cylindrical platinum electrode method, it has been demonstrated that in *A. calamus* the highest oxygen efflux occurs at the root apex and the amount decreases as one travels up the root [14]. However, the micro-sensor approach has not been applied to *A. calamus* growing in sediment. Accordingly, our study characterized the spatial patterns of rhizosphere oxygen profiles in single roots initiated at young stage and lasted over 3 weeks.

## Materials and Methods

### 1 Ethics statement

No specific permits were required for the described field studies, and the work did not involve any endangered or protected species (32°06′10.36″N, 118°54′13.75″E).

### 2 Plant material and culture conditions

In May 2012, overwintering tubers of *A. calamus* stored on a Water Environment Ecological Restoration Platform (NNU, Nanjing, China) were embedded in 25-L plastic containers containing 10 cm of silty sediment. The containers were filled up to a water table of 10 cm and placed in a glass greenhouse under natural lighting. After 3 weeks of pre-cultivation, seedlings with an approximate height of 12 cm were carefully removed from the sediment and transplanted into an experimental basin measuring 0.5×0.5 m. Intact roots (approx. 6 cm long) from three individual seedlings were carefully isolated and attached to a horizontal frame so that the roots could grew horizontally under the sediment at a fixed depth during the experimental period. Eutrophic mud was added to form 2 cm of sediment over the root, and the water table was adjusted to remain at 0.5 cm above the sediment. The experimental basin was incubated in a growth chamber under a 12-h dark/light photoperiod by SYLVANIA lamps (F24W/GRO, 400 µmol m^−2^ s^−1^ at the top of the canopy) with 28/22°C day/night temperature and 70/50% atmospheric humidity. Growth parameters, including shoot height (SH), blade width, root length, and root diameter (RD) were regularly recorded as the micro-optode measurements were taken.

### 3 Oxygen measurements

**Figure 1:**
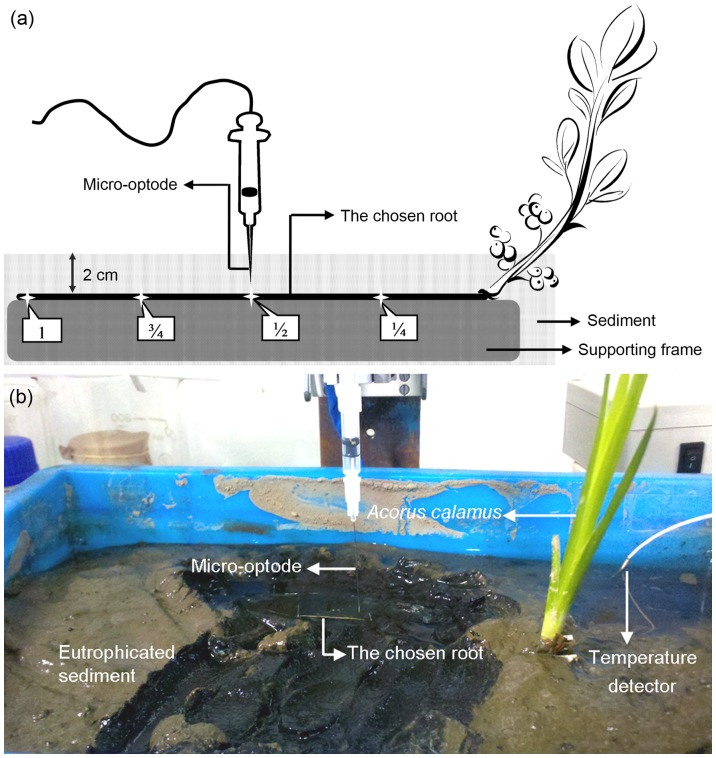
Schematic diagram (a) and an *in situ* monitoring photo (b) of the micro-optode system used for rhizosphere oxygen profiling. Selected intact roots (approx. 6 cm long) of *Acorus calamus* were carefully isolated and attached to a horizontal supporting frame. The internal and external oxygen saturations were determined using the oxygen microsensor system (Microx TX3 System, 25-µm probe; PreSens, Germany). The position in the root surface was determined partly from the signal and partly by visual inspection.

A micro-optode system (Microx TX3 System, 25-µm probe, PreSens, Regensburg, Germany) was used to measure the oxygen saturation in the rhizosphere and inside the roots. The Microx system consisted of a luminophore, an optical fiber as a signal transducer, and an optical sensor. The working principle and operation processes were detailed previously [27], [28]. As aqueous oxygen saturation depends on temperature, micro-optodes were calibrated separately before measurements with the help of a temperature detector buried in sediment (Fig. 1). One calibration value was obtained in a 1% Na_2_S_2_O_4_ solution (in the absence of oxygen), and the other was obtained in water vapor-saturated air. Each probe was used for approximately 1000–2000 single reads before it was recalibrated.

The measurements were performed at four locations on the root: one-quarter (1/4) of the way from the basal root (near the aboveground portion of the plant), halfway along the root (1/2), three-quarters of the way from the basal root (3/4), and at the apex or tip of the root (1) (Fig. 1a). First, silty sediment over the horizontally growing roots was temporarily removed from the measured range. The determination of the position in the root surface was made partly from the signal and partly by visual inspection (Fig. 1b). The microprobe was lifted and the root surface was gently covered with the original reduced sediment (2 cm) and readapted for 30 min before the determination was made [15]. To develop profiles of the oxic microzones, the microprobe was lifted vertically from the root surface with a micromanipulator in steps of 20 µm, and signals were recorded automatically. A mean of 30 reads within 30 s (each response  = 1 s) at each stop were calculated to determine the oxygen saturation, until the values declined to zero. The distance from the root surface to a stop at which 90% of root surface oxygen saturation remained was considered to be the depth of the stably oxidized microcosm [29]. The internal oxygen saturation was measured with a penetration of approximately 120–160 µm from the root surface into the aerenchyma on the basis of preliminary results.

Four measurements were carried out when the average shoot height reached approximately 15 cm (July 3, 2012), 30 cm (July 12), 45 cm (July 20), and 60 cm (July 27). At each measurement, three single roots were measured as triplicates.

### 4 Sediment

The silty sediment was collected from eutrophic Nanjing East City Lake (32°06′10.36″N, 118°54′13.75″E, water depth 1.2 m) in April 2012. The surface sediment was homogenized and wet-sieved (size fraction <2 mm) to remove debris and shells before used for cultivation of plants. Sediment used to readapt the roots during micro-optode measurements was sampled simultaneously with oxygen saturation measurements. Nutritional and physiochemical parameters of the sediment, including pH, soil redox potential (Eh), total nitrogen, total phosphorus, organic matter, porosity, and water content were determined on the same date that the rhizosphere oxygen saturation was determined respectively [30] (Table 1).

**Table 1:** Physicochemical parameters of sediment for *Acorus calamus* cultivation and readaptation to determine the rhizosphere oxygen saturation.

Shoot height	pH	Eh (mV)	TN (%)	TP (%)	OM (%)	Porosity (%)	WC (%)
15 cm	7.15	−187	0.39	0.15	3.99	76.56	54.11
30 cm	7.25	−185	0.40	0.16	4.00	75.81	53.64
45 cm	7.21	−186	0.37	0.15	3.95	75.47	53.37
60 cm	7.17	−192	0.37	0.15	3.92	76.23	53.89

TN: total nitrogen; TP: total phosphorus; OM: organic matter; WC: water content.

### 5 Chlorophyll fluorescence

DIVING-PAM (WALZ, Germany) was used to measure chlorophyll fluorescence parameters *in situ* in blades of *A. calamus*. Blades from three individual seedlings were randomly selected and the effective quantum yield of photosystem II, PSII, was measured in the middle to upper part, according to Bilger *et al*. [31]. Blades were dark-adapted for 15 min using clips before being exposed to 10 s of actinic light of 30 µmol m^−2^ s^−1^. A 0.8-s saturating pulse illumination with 4000 µmol m^−2^ s^−1^ was then generated to obtain the quantum yield.

### 6 Statistical analysis

Data were analyzed by a two-way ANOVA (using the relative position along the root and the growing stage as levels of classification) with SPSS software (SPSS 13.0 for Windows). The percentage values of oxygen saturation were arc-sin transformed to achieve normality and variance homogeneity. If the *F* value indicated significant differences (*p*<0.05), the means were compared using the Student–Newman–Keuls test with a confidence limit of 95%.

## Results

### 1 Plant growth and photosynthesis

**Figure 2:**
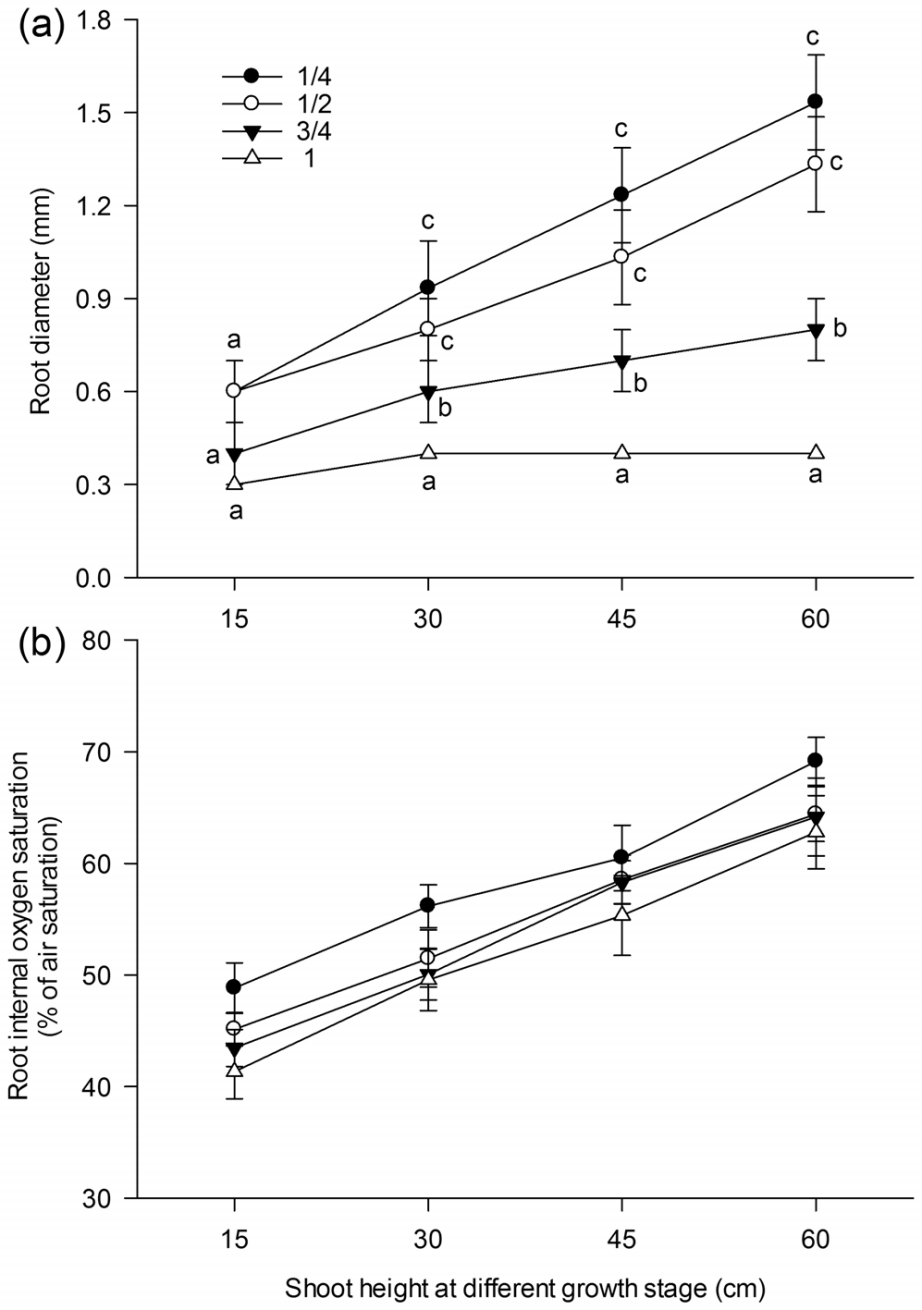
Root diameter and internal oxygen saturation in seedlings of *Acorus calamus*. The root diameters (a) were measured with a vernier caliper, and the internal oxygen saturations (b) were measured with a micro-optode at 1/4, 1/2, 3/4, and 1 (apex/root tip) along the entire root. Data points and vertical bars represent the mean of triplicates (each value was the mean of 30 reads from 3 roots of 3 seedlings each) and standard error, respectively. Different indices indicate that there is a significant difference according to the Students–Newman–Keuls test with a confidence limit of 95%. Note that the vertical scale is not the same for different positions.

Seedlings of *A. calamus* grew steadily without fluctuation in the effective quantum yield of PSII, which was 0.769 at the beginning of the experiment and 0.787 at the end (*p*>0.05). The average SH increased from 14.85 to 61.30 cm, and the average blade width increased from 0.47 to 1.21 cm. The average root length and RD (means of readings at the one-quarter, halfway, three-quarters, and apex positions along roots) increased from 5.9 to 11.2 cm and 0.5 to 1.0 mm, respectively. Root diameter significantly decreased from the basal part of the root to the apex (Fig. 2a) (*p*<0.05).

### 2 Internal oxygen supply

In all positions on the root, the internal oxygen saturation progressively increased as plants grew. For example, when SH increased from 30 to 45 cm, the average internal oxygen saturation (the mean of readings at the one-quarter, halfway, three-quarters, and apex position in roots) increased from 44.7% to 65.2%. However, no difference in internal oxygen saturation was observed among the four positions (Fig. 2b), indicating that the internal oxygen supply tended to be homogeneous from the basal part of the root to the apex during the entire growing stage.

### 3 Sediment oxygen profiles

**Figure 3:**
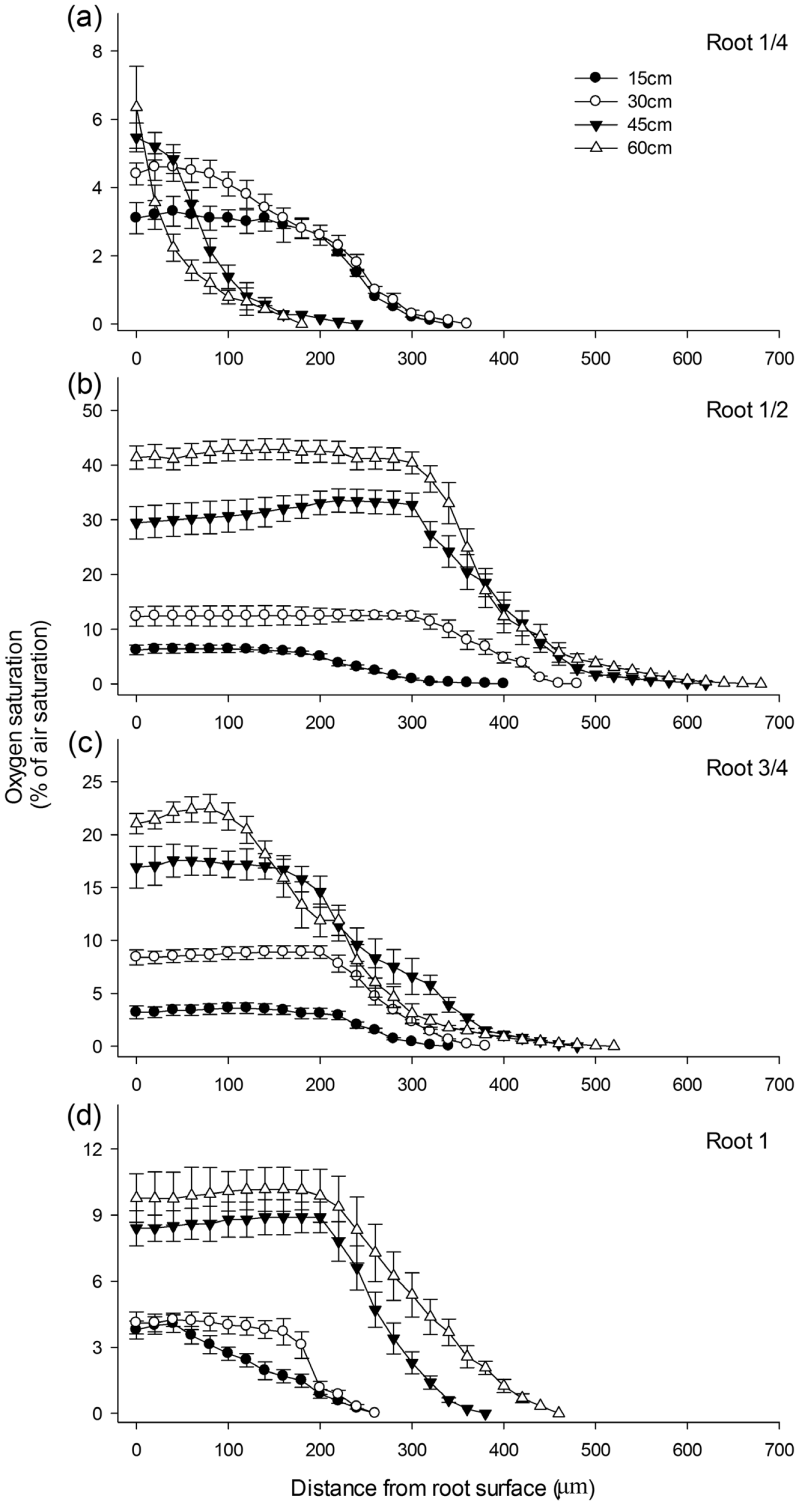
Rhizosphere oxygen saturation as *Acorus calamus* plants grow. Oxygen saturation was measured at 1/4 (a), 1/2 (b), 3/4 (c), and 1 (d, apex/root tip) along the entire root when the shoot height reached 15 cm, 30 cm, 45 cm, and 60 cm. Data points and vertical bars represent mean of triplicates (each value was the mean of 30 reads from 3 roots of 3 seedlings each) and standard error, respectively.

The physical and chemical properties of the sediment used for root readaptation were nearly unchanged during the experimental period, and thus their influence on oxygen saturation was assumed to be constant (Table 1). The oxygen profiles revealed the presence of an oxygen gradient from the root surface to the anaerobic bulk sediment at all growing stages (Fig. 3). As SH increased from 15 to 60 cm, the external oxygen saturation significantly increased at all positions except at the one-quarter point. External oxygen saturation was greatest at the point halfway along the root, followed by the three-quarter point and then the apex (Fig. 3a). As the plant grew, the maximum oxygen saturation at the halfway point increased from 6.4% to 42.9% (Fig. 3b), the oxygen saturation at the three-quarter point increased from 3.6% to 22.5% (Fig. 3c) and the oxygen saturation at the root apex increased from 4.1% to 10.2% (Fig. 3d). In contrast, only a small increase from 3.3% to 6.4% was observed in the maximum oxygen saturation at the one-quarter point.

**Figure 4:**
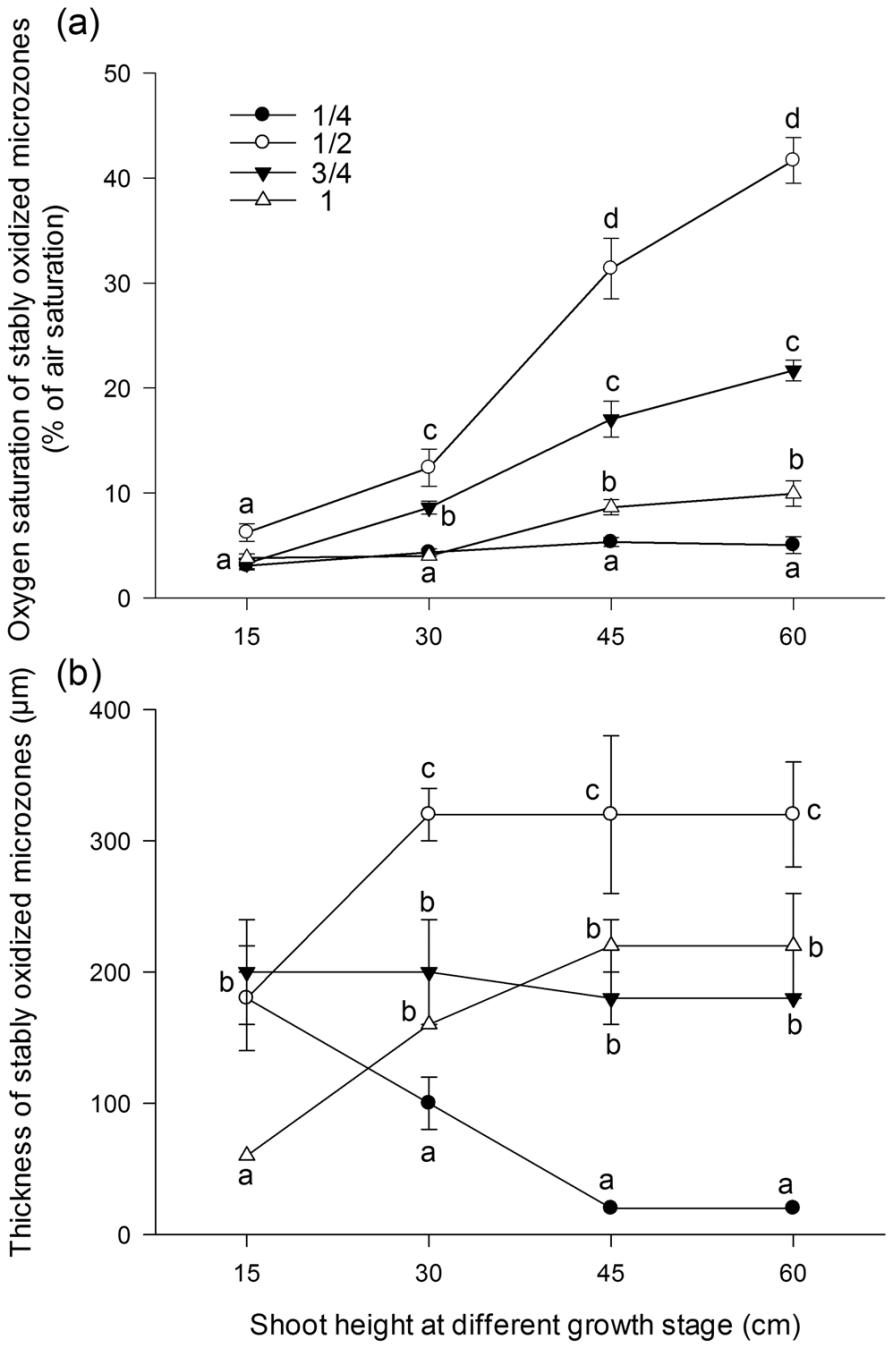
Oxygen saturation (a) and the thickness (b) of stably oxidized microzones in *Acorus calamus*. The distance from the root surface to a position maintaining 90% of root surface oxygen saturation is considered as the depth of the stably oxidized microcosm, according to Reddy et al. [29]. Data points and vertical bars represent the mean of triplicates and standard error, respectively (see Fig. 3). Different indices indicate significant differences according to the Students–Newman–Keuls test, with a confidence limit of 95%.

Oxygen saturation of the stably oxidized rhizosphere consistently peaked at the halfway point (Fig. 4a). In younger seedlings with a shoot height of approximately 15 cm, oxygen saturation remained below 10% for all positions and was roughly the same for all positions. When SH reached 30 cm, the oxygen saturation was greatest at the halfway point, followed by the three-quarter point, and the apex (*p*<0.05) and one-quarter point which were roughly equal (root 1/2> root 3/4> root 1≈ root 1/4). When SH reached 45 or 60 cm, oxygen saturation was greatest at the halfway point, followed by the three-quarter point, the apex, and the one-quarter point (root 1/2> root 3/4> root 1> root 1/4) (*p*<0.01).

### 4 Thickness of the oxidized rhizosphere

The oxygen diffusion zone (from root surface to anaerobic sediment) at the halfway point was approximately 320 µm when SH was 15 cm, 460 µm when SH was 30 cm, 580 µm when SH was 45 cm, and 620 µm when SH reached 60 cm. There were variations among the four positions in the thickness of the stably oxidized rhizosphere. The thickness decreased at the one-quarter point as plants grew, increased at the halfway point and at the apex, and remained nearly constant at the three-quarter point. The greatest thickness, 320 µm, appeared at the halfway point, whereas the smallest thickness was 20 µm and appeared at the one-quarter point (Fig. 4b). When SH was approximately 15 cm, the thickness of the stably oxidized rhizosphere was roughly the same for the three positions closer to the basal area, and was greater at these positions than at the apex (*p*<0.05). When SH exceeded 15 cm, the thickness was greatest at the halfway point, followed by the three-quarter point (*p*<0.05) and apex which were roughly equal, and then the one-quarter point (*p*<0.05). As SH increased from 30 to 45 cm, the thickness of the stably oxidized microzones at the one-quarter point declined from 0.10 to 0.02 mm.

## Discussion

### 1 Rhizosphere oxygen saturation and the thickness of stably oxidized microzones increase as roots grow

The level of rhizosphere oxygen saturation and the maintenance of stably oxidized microzones rely on continuous ROL. The factors determining ROL in aquatic plants include photosynthesis, stomatal gas exchange, and root morphology. During the day, photosynthetic oxygen is the dominant source [3], [9], but in the dark, gas exchange by stomata is the major source in the oxygen supply for ROL [32], [33]. The internal oxygen supply via aerenchyma affects shoot growth, as an increased leaf area for photosynthesis may produce a larger amount of oxygen.

**Figure 5:**
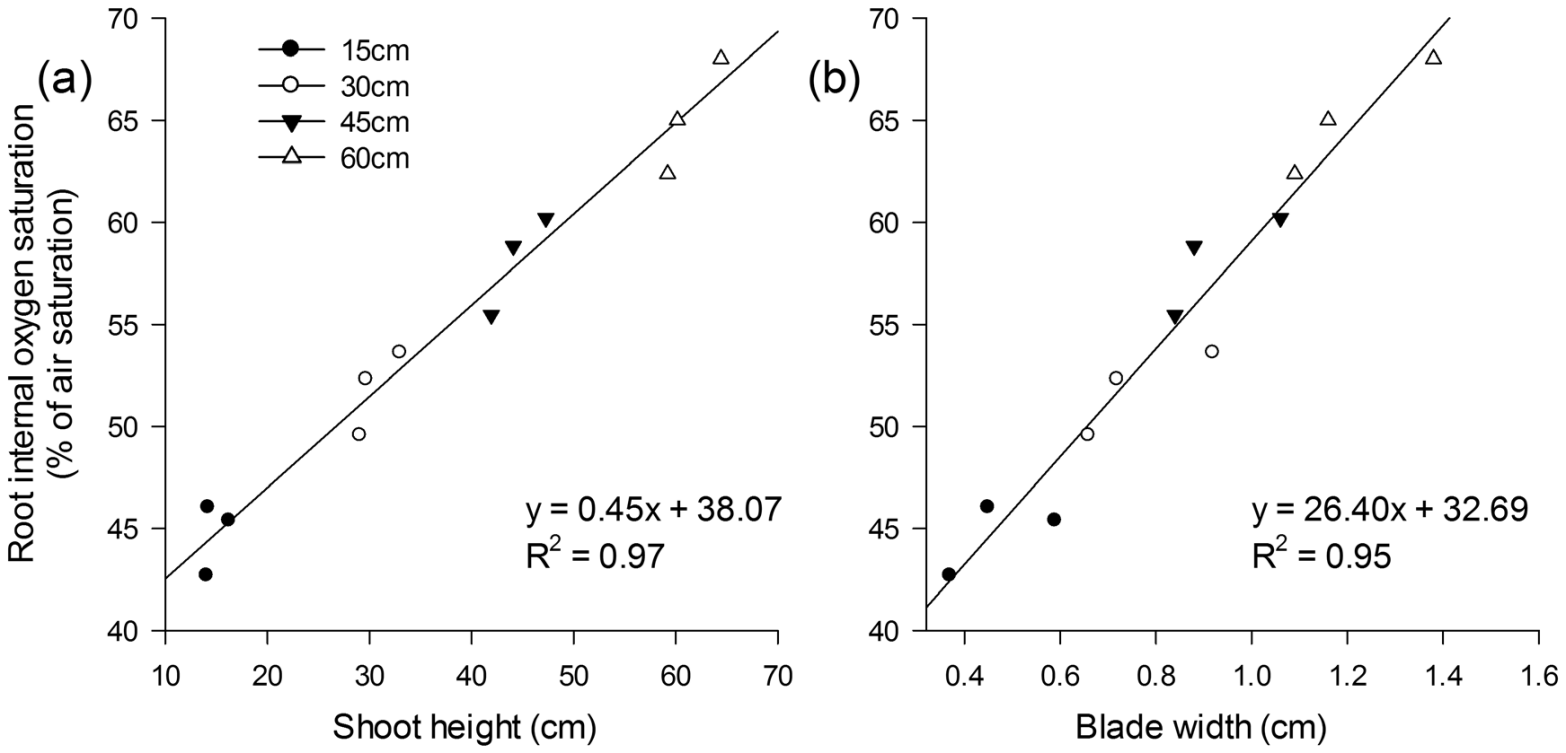
Correlation between the shoot height (a), the blade width (b), and the internal oxygen saturation. Values are the means of measurements at the 1/4, 1/2, 3/4, and 1 positions (apex/root tip) of the total root length from three single roots as triplicates.

**Figure 6:**
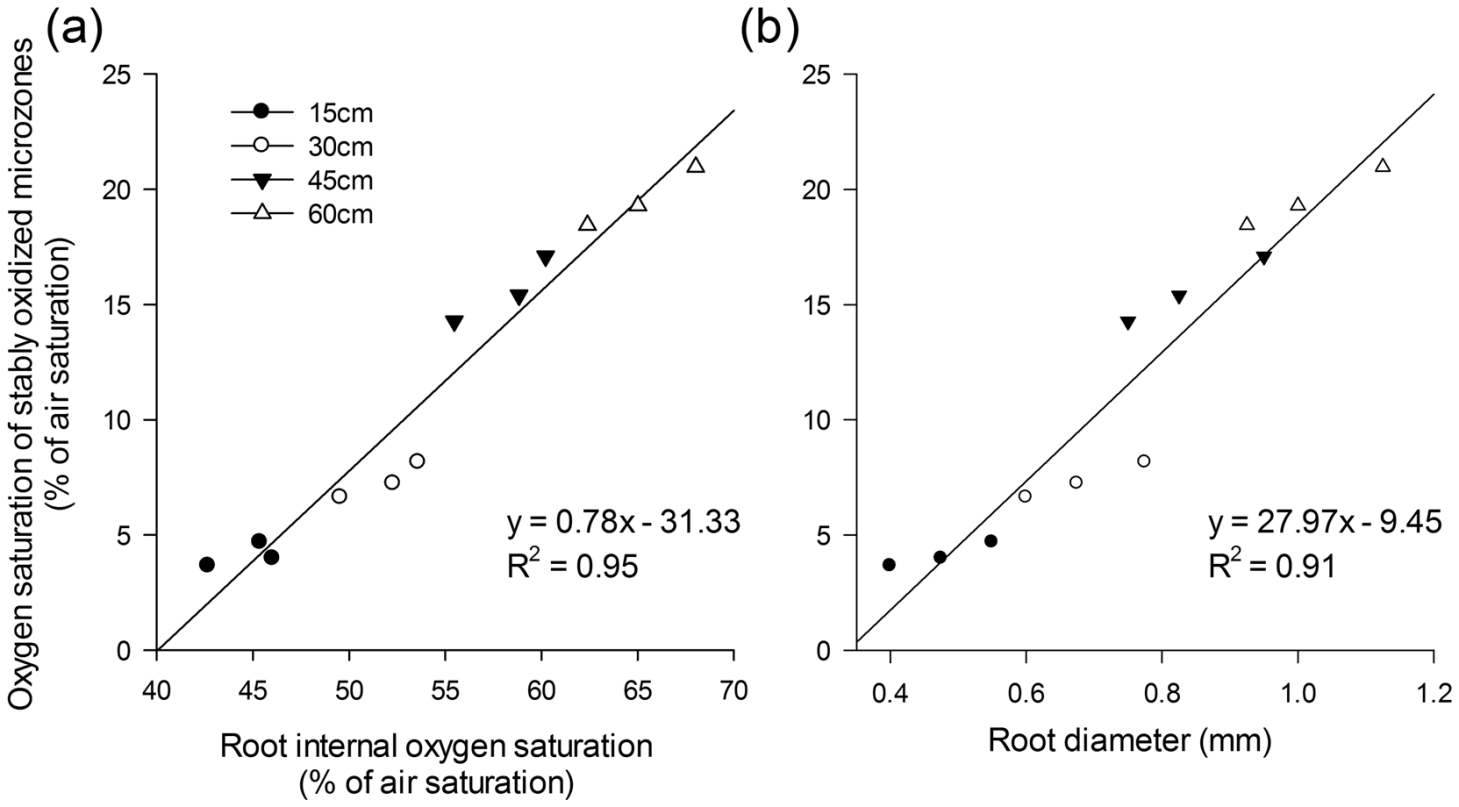
Root internal oxygen saturation (a), root diameter (b), and oxygen saturation of stably oxidized microzones. Values are the means of measurements at the 1/4, 1/2, 3/4, and 1 positions (apex/root tip) of the total root length from three single roots as triplicates.

In the present study, the effective quantum yield of PSII did not change during the entire experimental period (Table 2); therefore, the observed elevation in internal oxygen saturation is assumed to be induced by the increase in shoot height (Fig. 5a) and blade width (Fig. 5b). Porous root structures allow increased oxygen diffusion and can promote oxygen transport toward the root apex in *Potamogeton crispus* [15] and in selected lines of rice [13]. Aerenchyma are commonly well developed when seedlings of *A. calamus* growing [14] as in many other emergent plants [17]. The full development of aerenchyma and the increased oxygen-releasing area due to the increasing root diameter both contribute to the increase in oxygen efflux. The present study indicated that the actual rhizosphere oxygen saturation increased along with the increasing internal oxygen saturation (Fig. 6a) and root diameter (Fig. 6b).

**Table 2:** Vegetative growth and chlorophyll fluorescence parameters of *Acorus calamus* measured on the same date that the rhizosphere oxygen saturation was determined.

Growing stage (shoot height)	SH (cm)	BW (cm)	RL (cm)	RD (mm)	Y
15 cm	14.85±1.23	0.47±0.12	5.9±0.90	0.5±0.1	0.769±0.041
30 cm	30.62±2.12	0.77±0.15	9.5±1.50	0.7±0.1	0.772±0.034
45 cm	44.43±2.69	0.93±0.12	10.4±2.40	0.8±0.2	0.776±0.047
60 cm	61.30±2.78	1.21±0.18	11.2±2.20	1.0±0.2	0.787±0.025

Values represent means of triplicates and standard error, respectively. SH: shoot height; BW: blade width; RL: root length; RD: root diameter; Y: the effective quantum yield of PSII.

It has been reported that oxic microzones had a thickness of 80 µm in the seagrass *Cymodocea rotundata* using microeletrode aproach (detection limit 10 ppb) [17]. It has also been revealed that the oxidized rhizosphere of *Myriophyllum spicatum* and *P. crispus* was 100–250 µm thick [15]. In emergent plant species, the oxidized rhizosphere was reported to be less than 350 µm thick [34]. These results referred to an oxygen diffusion zone apart from the root surface to the complete anoxic sediment. Owing to the similarly high sensitivity of micro optode approach applying in the present study (detection limit 15 ppb), the thickness of the oxic microzones could be comapared with the above mentioned results using microeletrode aproach (approximately 10 ppb). In comparison, *A. calamus* generates a much pronounced oxidized rhizosphere; at the halfway point, the oxygen diffusion zone ranged from 320 to 620 µm around the root surface using micro optode aproach, which is 2 to 4 times greater than that in the submersed plants *M. spicatum* and *P. crispus* [15].

However, the present study demonstrated that in the outer boundary of the rhizosphere, away from the root surface, the oxygen level declined at various rates, depending on the relative position of the roots and the interfacial saturation on the root surface (Fig. 3). It is therefore worthwhile to estimate the stably oxidized zone in which an oxic microniche could be maintained. Translating the oxygen diffusion zones of *M. spicatum* and *P. crispus* reported previously [15] into stably oxidized microzones reveals that the thickness of oxygenated zone in *A. calamus* is 6 times that of *M. spicatum* and 10 times that of *P. crispus*. Although even greater oxic zones of 2.5–3.7 mm has been recorded in *Zostera marina* using the novel planar optode technique [4], the impermeable oxygen barrier of the glass plate in this technique unavoidably enlarged the observed oxic scale. Meanwhile, the oxygen depletion rate of different sediments may vary considerably, which also complicates a direct comparison between these thickness values.

**Figure 7:**
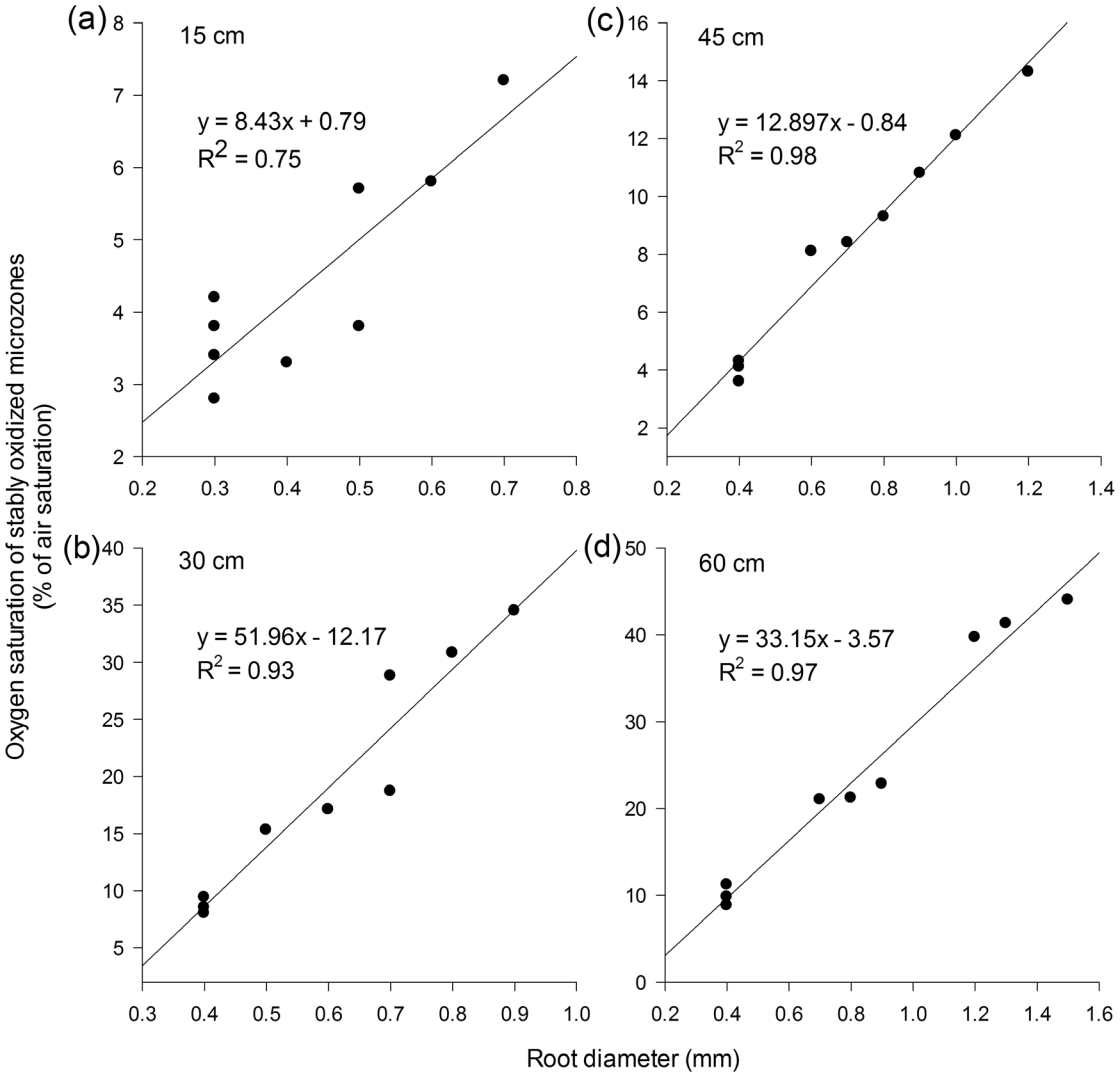
Correlation between the root diameter and the oxygen saturation of stably oxidized microzones. Oxygen saturation data at the 1/2 (b), 3/4 (a), and 1 (d) positions (apex/root tip) along the entire root were pooled at various growing stages with the shoot height of 15 cm (a), 30 cm (b), 45 cm (c), and 60 cm (d). Note that the vertical scale is not the same for the different positions.

Most of the literature refers to a narrow growing period and does not address the spatial evolution of rhizosphere oxygen profiles while roots growing. This study provides the first evidence in *A. calamus* that in younger seedlings (approximately 15 cm tall), there is no difference in the external oxygen saturation at different root positions, as long as the root diameter remains constant. However, when the shoot reached 30 cm or higher, the oxygen saturation in the basal root (the one-quarter point) declined below that of the middle position (the halfway point), in accordance with a similar trend in root diameter. As plants grew larger, the diameter of the root apex remained more or less constant, while that of higher positions increased progressively toward the basal parts of the root. With the exception of the one-quarter point, the correlation coefficients between oxygen saturation in the stably oxidized microzones and RD progressively increased (Fig. 7). Hence, the exclusive root positions (e.g., the distance to the basal root) with both minimal (one-quarter point) and maximal (halfway point) external oxygen saturation penetrate downward to deeper sediment. Therefore *A. calamus* oxygenates the rhizosphere and affects sedimentary biogeochemical processes starting at relatively young stage until the roots age and die back. On an ecosystem scale, emergent plant species thus exert great potential to absorb nutrients and immobilize toxic contaminants in deeper eutrophic sediments.

### 2 Rhizosphere oxygenation peaks around the middle part of the root

Three spatial patterns of ROL are documented in emergent plants: type I, in which oxygen saturation is relatively high and steadily decreases due to oxygen transport from the basal part to the root apex [12]; type II, in which the highest oxygen saturation appears in the root apical region and rapidly decreases toward the basal part; and type III, in which oxygen saturation is low in all root parts and remains nearly constant in different root parts [35]. A majority of emergent plants, including *Typha orientalis* [36] and *Rhizophora apiculata* [37], belong to type II. Using the cylindrical platinum electrode method within agar medium, it has been demonstrated that in *A. calamus*, the highest ROL occurs in the root apex and it decreases along the root upwards [14]; however, there has been no characterization of the *in situ* spatial pattern of rhizosphere oxygen profiles previously. ROL was defined as the measurements ratio of the total amount of released oxygen to the calculated total surface area of a single root, which estimated the average oxygen release of unit area of root surface [38]. Their estimation indeed indicated the net oxygen release by unit of root surface in anoxic artificial substrates. Rhizosphere oxygen saturation in sediment, however, vary among different root positions and is synergistically determined by ROL and RD [17].

In *A. Calamus*, it had also been shown that in anoxic eutrophic agar medium, the ROL in halfway point of roots was approximately 60% that of the apex [14]. In the present study, we recorded a nearly constant root diameter of around 0.3 mm in the root apex and a progressively increasing diameter at the halfway point of 3–4 times that of the apex (Fig. 2a). Consequently, the highest oxygen saturation appeared at the halfway point and it progressively decreased toward the root apex, and RD was significantly correlated with the oxygen saturation level in the stably oxidized microzones (Fig. 7), indicating that a higher RD could overtake the lowered ROL in the halfway point. In the submersed plants *C. rotundata* [17], *M. spicatum*, and *P. crispus* [15], the actual rhizosphere oxygen saturation from the basal part to the root apex was proportional to RD, in the agreement with our observations.

Interestingly, the greatest rhizosphere oxygenation in submersed plants occurred around the basal root in *M. spicatum*, and *P. Crispus* [15]. The ROL barrier and iron plaque can induce the decrease in the rhizosphere oxygen saturation around the basal roots [2], [22]. As an emergent plant species, *A. calamus* possesses long, fast-growing roots that are up to 35 cm in length and serve as the exclusive organ to absorb nutrients and fix the plant itself [39]. In contrast, roots in submersed plants such as *C. rotundata*, *M. spicatum*, and *P. crispus* are less than 12 cm long [15], [17]. To adapt to the enhanced length of the diffusion pathway in emergent plants, suberization and lignification occur in aerenchyma cells to form the “exodermis” barrier in the older part of roots; therefore, the root permeability and ability to release oxygen are greatly reduced in order to retain sufficient oxygen for the root apex [2], [3].

Meanwhile, when the *A. calamus* shoot reached or exceeded 30 cm, we observed a yellowish iron plaque that originated in the basal region of the root. In sediment, there is a simultaneous decrease in the actual rhizosphere oxygen saturation at the one-quarter point and in the other parts, confirming that the ROL barrier and iron plaque affect the oxygen profiles [40]. In contrast, the formation of the exodermis barrier or the accumulation of iron plaque were not apparatus in the younger seedlings of 15 cm, associated with a constant rhizosphere oxygenation around the entire root (unpublished data). Thus, the ROL barrier and iron plaque that accumulate on the older parts of the root surface modulate the distinctive spatial patterns of the rhizosphere oxygen profiles in *A. calamus*.

Although the noninvasive planar optode approach can produce *in situ* mapping of two-dimensional oxygen dynamics [4], [18], [19], [41], the resulting graphs demonstrate only partial roots that randomly pass through the analysis zone. The number, age, position of roots, and the interaction with other roots are not controlled. The temporal dynamics of oxygenation over a period that exceeds the length of time it takes for a single root to pass through the detection area (e.g., the weeks preceding maturation) are beyond the scope of study. The use of oxygen-impermeable glass plates also causes overestimation to the width of the stably oxidized microzones. To analyze the dynamics of rhizosphere oxygen profiles, semi-controlled growing single roots and minimally invasive measurements along the entire root provide a technical compromise. The present study suggests that the micro-optode approach is a feasible way to monitor the nearly natural rhizosphere oxygen profiles with minimal disturbance to roots and sediment.

## Conclusion

The present study is the first investigation using the micro-optode approach to examine the evolution of spatial patterns of rhizosphere oxygen profiles in *A. calamus*. It is interesting that the actual peak of rhizosphere oxygen saturation in sediment appears in the middle root and the thickness of the stably oxidized microzones are several times that of typical submersed plants. Rhizosphere oxygen saturation may be more closely associated with root diameter than with oxygen release from roots. In conclusion, the actual oxygen profiles in sediment differ from those in previous reports of ROL that applying conventional approaches and artificial substrates.
