# Correction: Spatio-Temporal Patterns in Rhizosphere Oxygen Profiles in the Emergent Plant Species *Acorus calamus*

**DOI:** 10.1371/journal.pone.0108084

**Published:** 2014-09-08

**Authors:** The PLOS ONE Staff

The authors’ first and last names are switched. Please view the correct author list, affiliations, and citation here:

Wenlin Wang^1,2^, Ruiming Han^1^, Yinjing Wan^3^, Bo Liu^1,4^, Xiaoyan Tang^2^, Bin Liang^2^, Guoxiang Wang^1^

**1** Jiangsu Key Laboratory of Environmental Change and Ecological Construction, College of Geographical Science, Nanjing Normal University, Nanjing, China

**2** Nanjing Institute of Environmental Sciences, Ministry of Environmental Protection, Nanjing, China

**3** Jiangsu Environmental Engineering Consulting Center, Nanjing, China

**4** School of Geography Science, Nantong University, Nantong, China

Wang WL, Han RM, Wan YJ, Liu B, Tang XY et al. (2014) Spatio-Temporal Patterns in Rhizosphere Oxygen Profiles in the Emergent Plant Species *Acorus calamus*. PLoS ONE 9(5): e98457. doi:10.1371/journal.pone.0098457
